# Centration axis in refractive surgery

**DOI:** 10.1186/s40662-015-0014-6

**Published:** 2015-02-24

**Authors:** Samuel Arba Mosquera, Shwetabh Verma, Colm McAlinden

**Affiliations:** SCHWIND eye-tech-solutions, Kleinostheim, Germany; Recognized Research Group in Optical Diagnostic Techniques, University of Valladolid, Valladolid, Spain; Department of Ophthalmology and Sciences of Vision, University of Oviedo, Oviedo, Spain; Flinders University, Adelaide, South Australia Australia; Wenzhou Medical University, Wenzhou, Zhejiang China

**Keywords:** Centration, Refractive surgery, Optical axis, Neural axis, Corneal vertex, Pupil centration, Corneal light reflex, Line of sight, Angle Kappa, Alpha, Lambda, Asymmetric offset

## Abstract

The human eye is an asymmetric optical system and the real cornea is not a rotationally symmetrical volume. Each optical element in the eye has its own optical and neural axes. Defining the optimum center for laser ablation is difficult with many available approaches. We explain the various centration approaches (based on these reference axes) in refractive surgery and review their clinical outcomes. The line-of-sight (LOS) (the line joining the entrance pupil center with the fixation point) is often the recommended reference axis for representing wavefront aberrations of the whole eye (derived from the definition of chief ray in geometrical optics); however pupil centration can be unstable and change with the pupil size. The corneal vertex (CV) represents a stable preferable morphologic reference which is the best approximate for alignment to the visual axis. However, the corneal light reflex can be considered as non-constant, but dependent on the direction of gaze of the eye with respect to the light source. A compromise between the pupil and CV centered ablations is seen in the form of an asymmetric offset where the manifest refraction is referenced to the CV while the higher order aberrations are referenced to the pupil center. There is a need for a flexible choice of centration in excimer laser systems to design customized and non-customized treatments optimally.

## Introduction

The human eye is an optical system comprising four main non coaxial optical elements (anterior and posterior corneal and lens surfaces), an aperture stop (pupil) and an imaging film in the form of a light sensitive tissue layer called the retina, but conforming a robust aplanatic design compensating the spherical aberrations and coma through non-planar geometry. Each optical element has its own optical (axis containing the center of curvatures of the optical surfaces of the eye) and neural axes (axis of receptors and retinal neurons peaking at the foveola and declining monotonically with increasing eccentricity). Although, the optical surfaces are aligned almost co-axially, the deviations from a perfect optical alignment results in a range of optical and neural axes and their inter relationships. The sharpest vision of a target is realized when it is in line with the fixation target and the fovea of the retina (visual axis). Displacing the pupil or the target object from this axis results in reducing the optical and visual properties of the system. In this literature review, we summarize the optical and neural axes of the eye along with their interrelationships. Further, we present a perspective on the difference between the on and off axis performance of the eye in terms of the optical and neural image quality. These metrics significantly affect the performance and outcomes of popular laser based refractive surgeries [1]. Therefore, we discuss their implications in context of centration axis in refractive surgery.

## Review

### Optical and neural axes of the eye

In the history of physiological optics, many axes of the eye have been described with conflicting and confusing definitions. We follow the definitions presented by Thibos et al. [2]. Other schematic representations of the different axes can be found here [3,4].

#### Optical axis

**Figure 1:**
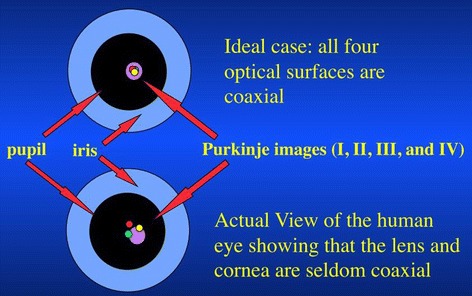
**Purkinje images of the human eye compared to an ideal coaxial optical system.** (Image courtesy of: Thibos LN: How to Measure Chromatic Aberration and Locate Useful Reference Axes of the Human Eye - OSA conference 1995; Portland. Published with permission from the author).

It is defined as the axis containing the center of curvatures of the optical surfaces of the eye. The optical axis can be determined when the reflecting virtual image of a point source lies between the object and the reflecting surface center. If the optical surfaces of the eye were perfectly coaxial, the reflected images from each optical surface would appear aligned from the perspective of an object that is positioned on the optical axis. The Purkinje images (I, II, III, and IV) are the reflections of objects from the structures of the eye, namely the outer corneal surface (I), inner corneal surface (II), anterior surface of the lens (III) and the posterior surface of the lens (IV) respectively. These images are however seldom observed to be coaxial showing deviations from an ideal coaxial optical system (Figure 1).

#### Visual axis

**Figure 2:**
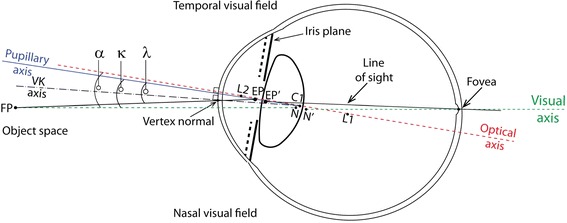
**Schematic sketch of the reference angles and axes in the human eye.** The axes are indicated by the following lines; solid black (line of sight), solid blue (pupillary axis), dashed green (visual axis), dashed red (optical axis), and dashed black (videokeratoscope axis). The centers of curvature of each refracting surface are represented as L2, C2, C1, and L1. (Reprinted from Biomedical Optics Express, Vol. 3, Issue 2, Nowakowski M, Sheehan M, Neal D, Goncharov AV, Investigation of the isoplanatic patch and wavefront aberration along the pupillary axis compared to the line of sight in the eyem, Pages 240–258, Copyright © 2012 The Optical Society All Rights Reserved, published with permission of The Optical Society.).

It is defined as the line connecting the fixation point with the foveola, passing through the two nodal points of the eye represented by N and N’ in Figure 2. The two nodal points coincide at the center of curvature of the surface such that the slope of the ray directed towards the first nodal point is the same as the slope of the ray that appears to emerge from the second nodal point. A ray that is normal to an optical surface will pass undeviated through the nodal point. This nodal ray will therefore, exhibit zero transverse chromatic aberration (TCA). Hence, the visual axis can be determined as the nodal ray that strikes the foveola with zero TCA. For this reason, the visual axis is also called as the foveal achromatic axis. The visual axis does not necessarily pass through the pupil center (PC), and can be imagined as a straight line from fixation point to foveola (with the patient fixating), representing an undeviated or minimally deviated ray of light.

#### Pupillary axis

It is defined as the normal line to the corneal surface that passes through the center of the entrance pupil and the center of curvature of the anterior corneal surface. The PC can be observed directly. Pupillary axis can be determined locating a source such that the reflected image of this source (when viewed from the source) is centered on the entrance pupil.

#### Line of sight

It is defined as the ray from the fixation point reaching the foveola via the PC. The line of sight (LOS) is slightly different in the object and image plane of the eye. In general, it can be imagined as a broken line representing a deviated ray of light, going from the fixation point to the PC (with the patient fixating) and eventually reaching the foveola after refraction at each optical interface. The LOS is associated with a comparatively longer optical path difference (OPD) compared to visual axis, also showing TCA unlike the visual axis. It can be determined using two point sources at different distances from the eye fixated simultaneously, one focused on the retina and one out-of-focus. If the chief rays from both sources are coincident and they lie on the LOS, the ray from the out-of-focus source shall form a blur circle while the ray through PC (focused source) shall form the center of the blur circle.

#### Achromatic axis

It is defined as the axis joining the PC and nodal points. A chief ray from an object on this axis shall have zero TCA. The peripheral retina (outside the fovea) is affected by poor spatial resolution. Hence, it is difficult to locate the eccentricity of the achromatic axis. Conversely, the separation between the PC and visual axis can be used to quantify the eccentricity at which targets are imaged without any TCA.

#### Photoreceptor axis (peak of the Stiles Crawford effect)

Humans are more sensitive to light passing through the section of the pupil that is coaxial with the receptor axis from the retina. Hence, the pupil appears to be apodized (called the Stiles Crawford effect). Narrow beams projected through different pupil locations are used to calculate the Stiles Crawford function [plot of contrast sensitivity (CS) versus pupil location]. The peak of this function is used to locate the photoreceptor axis.

#### Neural axis

The spatial bandwidth of the veridical neural image peaks at the foveola and declines monotonically with increasing eccentricity. The neural axis can be determined by locating the spatial frequency at which veridical perception of a grating becomes aliased.

#### Angle between the optical and neural axes

Angle Alpha: Angle formed at the first nodal point by the eye’s optical and visual axes.

Dunne et al. [5] tested the association between peripheral astigmatic asymmetry and angle alpha in 34 eyes. Their results indicate that either peripheral astigmatic asymmetry is due to additional factors such as lack of symmetry in the peripheral curvature of individual optical surfaces or there is further misalignment of optical surfaces away from an optical axis.

Angle Kappa: Angle between pupillary and visual axes.

Hashemi et al. [6] determined the mean angle kappa and its determinants in the population of Tehran, Iran, in a cross-sectional survey with random cluster sampling and a total of 442 participants aged >14 years. Mean angle kappa was 5.46 ± 1.33° in total; 5.41 ± 1.32° in men and 5.49 ± 1.34° in women (P = 0.558). It decreased significantly with age; 0.015°/year (P < 0.001). In individuals with myopia, emmetropia, and hypermetropia, the mean value was 5.13 ± 1.50°, 5.72 ± 1.10°, and 5.52 ± 1.19° respectively (P = 0.025); the post-hoc test indicated this was due to the difference between emmetropes and myopes. They concluded that angle kappa reduced with age, and the inter-gender difference was not significant. Largest angle kappas were seen among individuals with emmetropia. Angle kappas were larger in the hypermetropic population compared to the myopic population. In a similar study performed to investigate the normative angle kappa data and demographic features in Koreans [7], angle kappa decreased with axial length and increased with age and spherical equivalent. Giovanni et al. [8] suggested that emmetropes and hypermetropes tend to have a larger angle kappa than myopes. Basmak et al. [9] also reported that the angle kappa decreases as the refractive error becomes more negative. They speculated that the corneal intercepts of the axes were located closer to the optical axis in myopic eyes and farther away in hyperopic eyes. The differences in these results could be attributed to the ethnic variations in ocular anatomy [10]. A statistically larger interpupillary distance may influence the angle kappa as observed in a comparative study with African-American and white patients [11].

Angle Lambda: Angle between pupillary axis and the LOS.

Lu F et al. [12] measured the horizontal coma in the anterior cornea, the whole eye, and the internal optics for 221 young subjects. Thirty-three eyes with minimum angle lambda and 53 eyes with relatively large angle lambda were selected from these eyes to test the hypothesis that horizontal coma compensation is linked to angle kappa. Significant horizontal coma in the anterior cornea was observed for the group with minimum angle lambda in both the right (−0.12 ± 0.07 μm) and left eyes (0.12 ± 0.10 μm), and this was well compensated by the internal optics, so that the level of horizontal coma in the whole eye over a 6-mm pupil size was very low (−0.05 ± 0.07 μm for OD and 0.02 ± 0.08 μm for OS).

Salmon et al. [13] explored the effect of the difference in the reference axis used in videokeratoscopy and Shack-Hartmann aberrometry. The Shack-Hartmann aberrometer is usually aligned coaxially with the LOS (PC), but videokeratoscopes usually are not. They developed a method to compensate for videokeratoscope-LOS misalignment, and analyzed the importance of compensating for the misalignment. Their results show that when the value of angle lambda (the angle between the LOS and the pupillary axis) is larger than 2–3 degrees, the misalignment, if ignored, can lead to incorrect estimates of corneal and internal aberrations as well as corneal/internal aberration balance.

The various reference axes and angles are presented in the Figure 2.

### On and off axis visual performance

Decentration of the entrance pupil can introduce a variety of optical aberrations such as TCA, coma, and astigmatism. Green [14] measured CS for sinusoidal gratings presented on an oscilloscope as a function of the location of a small (2 mm) artificial pupil. He found that decentration of the pupil led to large decreases in visual acuity (VA) and an even larger decline in mid- and high-frequency CS. Green attributed the loss in CS observed in the normal incoherent experiment to coma caused by off-axis viewing in an eye with spherical aberration. Van Meeteren and Dunnewold [15] and Thibos [16] both argued that the ocular chromatic aberration (and not spherical aberration or coma) were responsible for the reduction in CS and VA with pupil decentration. Finally, Campbell [17], and Campbell and Gregory [18] argued that reduced VA for decentered ray-bundles could be explained by the anatomical properties of the photoreceptors. Schematic eye models have been designed to simulate off-axis aberrations at wide angles [19-21]. The aberrations of the cornea are partially compensated by the aberrations of the internal optics of the eye (primarily the crystalline lens) in young subjects. Marcos et al. [22] investigated the active or passive nature of the horizontal coma compensation using eyes with artificial lenses where no active developmental process can be present. On average, they found that spherical aberration was compensated by 66%, and horizontal coma by 87%. The fact that corneal (but not total) horizontal coma is highly correlated with angle lambda (computed from the shift of the 1st Purkinje image from the PC, for foveal fixation) indicates that the compensation arises primarily from the geometrical configuration of the eye (that generates horizontal coma of opposite signs in the cornea and internal optics) [23].

### Centration in refractive surgery

The centration of ablation in refractive surgery has been extensively studied. Different centration approaches are applied by commercial laser systems used in refractive surgery (Table 1). A decentered ablation results in an eccentric optical zone (OZ) with the patients complaining of quality of vision issues such as nighttime glare [24-26]. Controversy remains regarding optimal centration in corneal refractive procedures. The ideal location to maximize visual outcome is yet to be determined. However, Reinstein et al. [27] determined whether centering ablations on the coaxially sighted corneal light reflex (CSCLR) in eyes with large angle kappa leads to poor visual outcomes when compared to patients with eyes possessing small angle kappa that by default would be centered on the entrance pupil. Eyes were divided into two discrete groups according to the pupil offset: small angle kappa for pupil offset of 0.25 mm or less (n = 30) and large angle kappa for pupil offset of 0.55 mm or greater (n = 30). They found no statistically significant differences in safety, accuracy, induced astigmatism, CS, or night vision disturbances between the two groups.

**Table 1:** **A summary of the centration techniques applied by various commercial laser refractive systems**

**S. No.**	**Company**	**Device**	**Technique**	**Applied**	**Type**
**1**	Alcon	LadarVision 6000	Semi-Automated based on on-screen identification of CSCLR	Under the laser	The whole ablation is shifted
**2**	Bausch & Lomb	217 Zyoptix	Manually based CLR (but not truly CS)	Under the laser	The whole ablation is shifted
**3**	Bausch & Lomb	317 Teneo	Manually based CLR (but not truly CS)	Under the laser	The whole ablation is shifted
**4**	CustomVis	Pulzar Z1	Fully-Automated based on limbus registration	Under the laser	The whole ablation is shifted
**5**	iVIS	iRES	Fully-Automated based on iris registration	Under the laser	The whole ablation is shifted
**6**	KATANA	LaserSoft	Manually based CLR (but not truly CS)	Under the laser	The whole ablation is shifted
**7**	KERA	IsoBeam	Manually based CLR (but not truly CS)	Under the laser	The whole ablation is shifted
**8**	LaserSight	AstraScan	Manually based CLR (but not truly CS)	Under the laser	The whole ablation is shifted
**9**	Nidek	Quest	Manually based CLR (but not truly CS)	Under the laser	The whole ablation is shifted
**10**	Novatec	LightBlade	Manually based CLR (but not truly CS)	Under the laser	The whole ablation is shifted
**11**	SCHWIND	ESIRIS	Manually based on Corneal Vertex (numerically taken from diagnosis)	Under the laser	The whole ablation is shifted
**12**	SCHWIND	AMARIS	Manually based on Corneal Vertex (numerically taken from diagnosis)	During treatment planning	Only the optical axis is shifted (even for customized treatments), but the whole ablation remains concentric to the pupil boundaries
		AMARIS 500E			
		AMARIS 750S			
		AMARIS 1050RS			
**16**	VISX	Star S4 IR	Fully-Automated based on iris registration	Under the laser	Only pupil centration is possible
**17**	WaveLight	Allegretto	Manually based CLR (but not truly CS), for large offsets or angles (alpha, kappa, lambda) “in between”	Under the laser	The whole ablation is shifted
		Allegretto-Eye-Q			
		EX500			
		Concept1000			
**18**	ZEISS-Meditec	MEL80	Manually based CLR (but not truly CS), considering contralateral viewing eye to reduce parallax	Under the laser	The whole ablation is shifted
		MEL90			

CS: coaxially sighted; CLR: corneal light reflex; CSCLR: coaxially sighted corneal light reflex. It is worth noting that iVIS iRES, KATANA LaserSoft, KERA IsoBeam, LaserSight AstraScan, Nidek Quest, SCHWIND AMARIS, WaveLight Allegretto and EX500, and ZEiSS-Meditec MEL80 and MEL90 use a video based eye-tracker from the same supplier in slightly different variations.

We present below some recent studies evaluating and comparing the centration references in refractive surgery.

#### Corneal light reflex

The corneal light reflex is formed by the reflection of light from the anterior corneal surface. In other words, the virtual image of the light source which is also known as the first Purkinje-Sanson image. Many researchers have postulated that the coaxial light reflex from the cornea lies closer to the corneal intercept of the visual axis than the PC and thus recommend the corneal coaxial light reflex as the center in refractive surgery [27].

Pande and Hillmann [3] studied the differences in OZ marking using the geometric corneal center, entrance PC, visual axis, and the coaxially sighted corneal reflex as centration points. They used a modified autokeratometer to photograph the cornea in 50 volunteers under standardized levels of illumination, with the subject fixating on the keratometer target. They marked the above-mentioned centration points and measured the direction and degree of decentration. They found that from the corneal intercept of the visual axis, the entrance PC was up to 0.75 mm (0.34 ± 0.20 mm) temporally, the corneal reflex was found up to 0.62 mm (0.21 ± 0.16 mm) nasally, and the geometric corneal center was found up to 1.06 mm (0.55 ± 0.22 mm) temporally. Based on these decentration measurements they concluded that the corneal light reflex was the nearest point to the corneal intercept of the visual axis. In the absence of an offset, i.e. null angle alpha, kappa and lambda; PC, CV, CSCLR and visual axis groups shall all collapse into one. However, with the naturally occurring offset angles, determination of the closest corneal intercept of the visual axis is imperative for precise ablation centration.

Nepomuceno et al. [28] analyzed the VA, CS, and target deviations in 37 consecutive patients (61 eyes) who had laser in situ keratomileusis [LASIK, LADARVision - 4000 excimer laser (Alcon)] for primary hyperopia with the ablation centered on the CSCLR. CS log units were measured using the CSV-1000 CS chart (Vector Vision) at a spatial frequency of 12 cycles/degree (cpd). Postoperatively, the uncorrected VA was 20/20 or better in 44.4% of eyes. The mean deviation from the target refraction was +0.25 diopters (D) ± 0.82 (SD), with 65.6% of eyes within ±0.50 D of target. No eye lost 2 or more lines of best corrected VA (BCVA). A loss of 3 or more patches of best spectacle-corrected contrast sensitivity (BSCCS) was seen in 6.6% of the eyes and a loss of 4 or more patches, in 1.6%. Ablation zone centered on the CSCLR did not adversely affect BCVA or BSCCS.

Chan et al. [29] analyzed the postoperative topographic centration when the CSCLR was used for laser centration in 21 eyes (12 patients) that underwent hyperopic LASIK using LADARVision 4000 (Alcon Laboratories, TX, USA). The mean deviation of the CSCLR from the entrance PC preoperatively was 0.34 ± 0.24 mm nasal or 4.5 ± 3.0 degrees. At 1 day, the average decentration was 0.10 mm or 1.3 degrees temporal. The mean decentration that would have occurred if the ablation had been centered over the entrance PC was 0.44 mm or 5.5 degrees temporal. At 3 months, the average decentration was 0.07 mm or 0.25 degrees temporal. The mean decentration that would have occurred if the ablation had been centered over the entrance PC was 0.45 mm or 5.6 degrees temporal. Mean uncorrected VA (log MAR) improved 3 lines from 0.54 ± 0.14 (20/70) to 0.22 ± 0.17 (20/32). No eye lost >2 lines of BCVA; 2 (10%) eyes lost 1 line of BCVA at 3-month follow-up. They concluded that excellent centration in hyperopic ablation is possible even in eyes with positive angle kappa when the ablation is centered over the corneal light reflex.

The entrance pupil is a virtual image formed by the light reflex from the real pupil refracted by the cornea. The corneal light reflex can be considered as non-constant but this is dependent on the direction of gaze of the eye with respect to the light source. An examiner behind the light source can observe the deviation in corneal light reflex as the direction of gaze changes. Furthermore, due to the parallax between the entrance pupil and the corneal light reflex, the exact projection of the corneal light reflex on to the patient entrance pupil depends on the position of the examiners eye behind the light source. The CSCLR will be seen differently depending on the surgeon’s eye dominance, surgeon’s eye balance, or the stereopsis angle of the microscope. In order to avoid these complications, other centration approaches are also preferred by some researchers.

#### Line of sight (pupil centration)

PC considered for a patient who fixates properly defines the LOS in refractive procedures. Uozato and Guyton [30] obtained the best optical result by centering the surgical procedure on the LOS and entrance pupil of the eye, not on the visual axis. They found an error of 0.5-0.8 mm when referencing the visual axis, which probably arose from the use of corneal light reflex as a sighting point or from inadvertent monocular sighting in techniques requiring binocular sighting. They explained that for an ideal centration, the patient should fixate at a point that is coaxial with the surgeon’s sighting eye and the cornea is marked with the center of the patient’s entrance pupil ignoring the corneal light reflex. They concluded that for the best optical results, the procedure must be centered on the LOS and the entrance pupil of the eye.

Artal et al. [31] stated that the position of the pupil is important for the correct estimation of retinal image quality and should be taken into account when predicting visual performance from corneal aberration data. Marcos et al. [32] evaluated the optical aberrations induced by LASIK refractive surgery for myopia on the anterior surface of the cornea and the entire optical system of the eye. They measured the total wavefront aberrations using a laser ray tracing with a reference to pupil centration. The corneal wavefront aberrations were calculated from the corneal elevation (with corneal reflex centration) centered at −0.6 to +0.6 mm from the corneal reflex. This was done to maintain comparable centration reference between the corneal and total aberrations at the PC. The PC was found typically, slightly decentered from the corneal reflex. Apart from the decentration between the corneal reflex and PC, the keratometric axis is tilted with respect to the LOS. This angle can be computed by measuring the distances between the corneal intersect of the keratometric axis and corneal sighting center. According to their computations, corneal aberration data (third-order and higher) changed by 10% when the pupil position was taken into account. Spherical aberration did not change significantly by recentration (3% on average), while third-order aberrations changed by 22%.

Another approach for ablation centration could be to focus on the presumed photoreceptor axis. Since the photoreceptors are aimed at the center of the pupil, light passing through the center of the normal pupil is more effective in simulating photoreceptors. This argument reinforces the use of pupil centration as reference. However, referencing the photoreceptor axes directly or indirectly has not been studied clinically.

#### Visual axis (normal corneal vertex centration)

**Figure 3:**
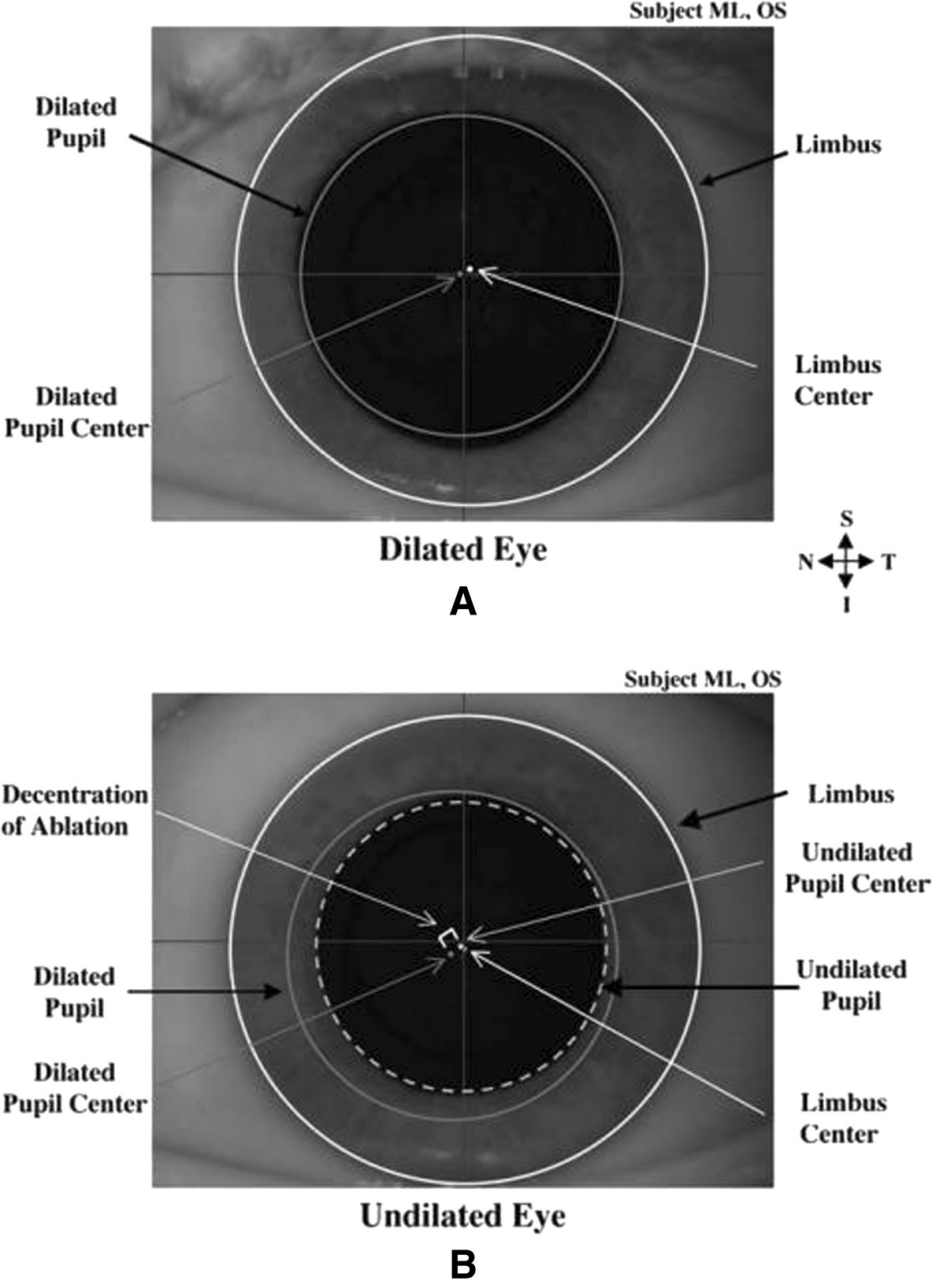
**Images of the same left eye in pharmalogically and naturally dilated states.** Here **(A)** represents pharmacologically dilated state (Neo-Synephrine 2.5%) and **(B)** represents natural undilated state. The edges of the limbus and dilated pupil are illustrated using solid white and solid dark gray lines respectively, while that of the undilated pupil is denoted using a dashed light gray line. Limbus, dilated pupil, and undilated PCs are represented by white, dark gray, and light gray circles, respectively. A customized ablation in this eye could be decentered due to a slight superotemporal shift from when aberrations were measured over a dilated pupil to when they were corrected over an undilated pupil. (Reprinted from J Cataract Refract Surg, Vol 32, Issue 1, Porter J, Yoon G, Lozano D, Wolfing J, Tumbar R, Macrae S, Cox IG, Williams DR, Aberrations induced in wavefront-guided laser refractive surgery due to shifts between natural and dilated pupil center locations, Pages 21–32, Copyright © 2006. published with permission from Elsevier.).

**Figure 4:**
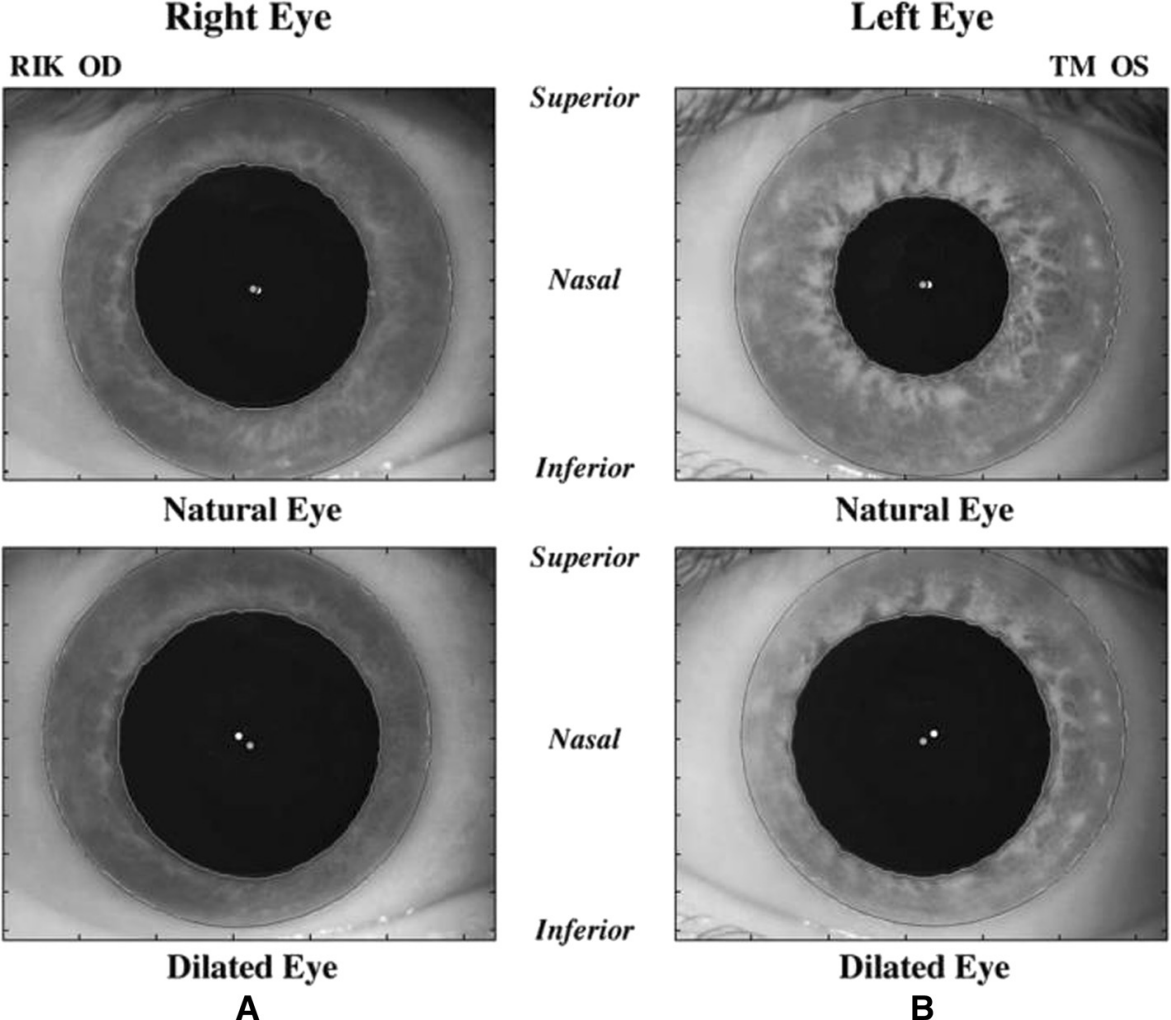
**Changes in pupil center location and iris shape with pupil dilation.** These images illustrate the change in pupil center location and iris shape from a natural undilated state to a dilated state in **(A)** one patient’s right eye and **(B)** a different patient’s left eye. Superior, nasal, and inferior directions are noted on the figure. White and gray filled circles denote limbus and pupil centers, respectively. Irises tended to thin more in the inferonasal direction than in the superotemporal direction. Pupil centers tended to shift in the inferonasal direction with dilation. (Reprinted from J Cataract Refract Surg, Vol 32, Issue 1, Porter J, Yoon G, Lozano D, Wolfing J, Tumbar R, Macrae S, Cox IG, Williams DR, Aberrations induced in wavefront-guided laser refractive surgery due to shifts between natural and dilated pupil center locations, Pages 21–32, Copyright © 2006. published with permission from Elsevier.).

**Figure 5:**
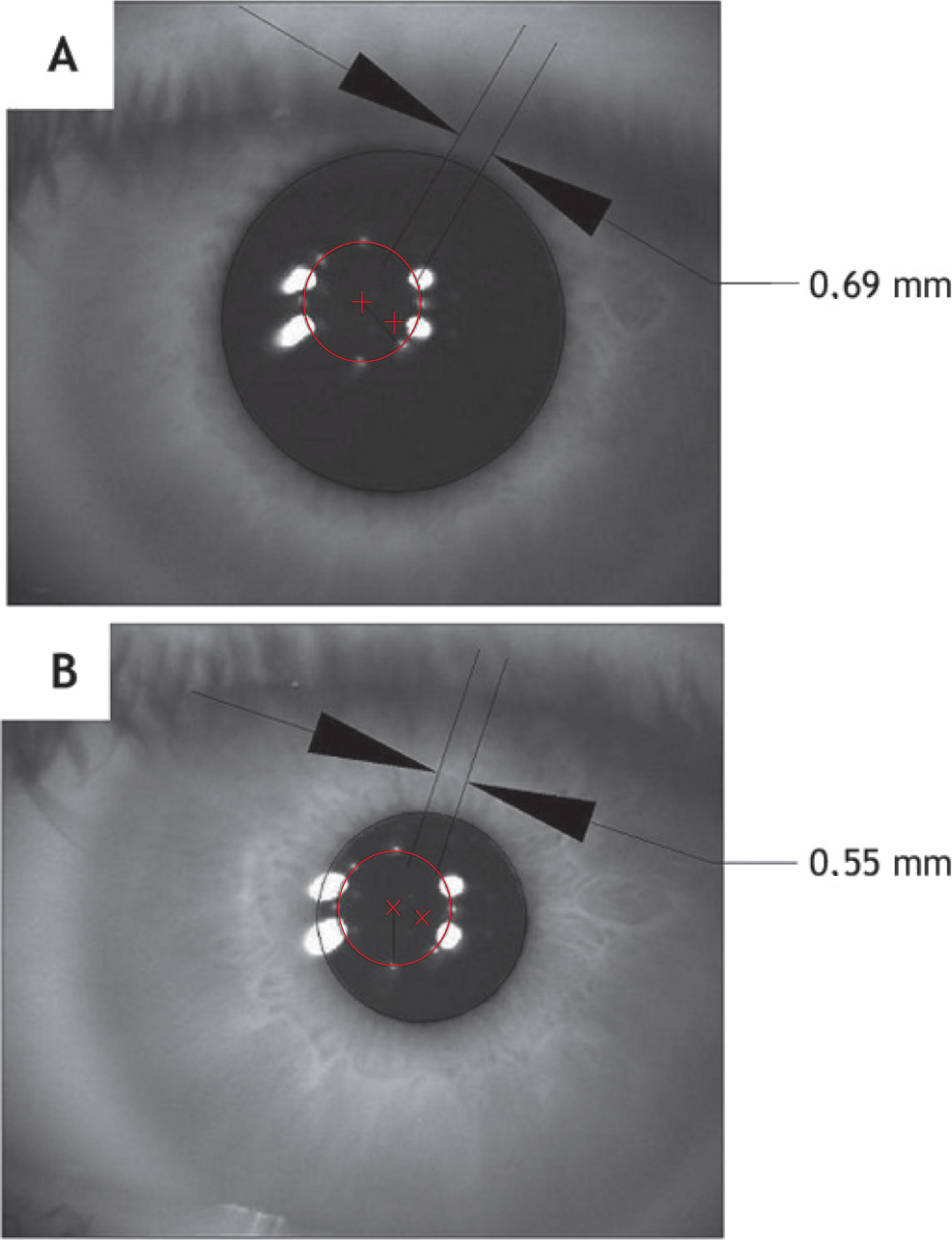
**Images of the pupil center for low (A) and high (B) lighting conditions.** Pupil decentering values are included for both conditions for comparison. (Reprinted from Journal of Optometry, Vol 4, Issue 4, Montés-Micó R, Hernández P, Fernández-Sánchez V, Bonaque S, Lara F, López-Gil N, Changes of the eye optics after iris constriction, Pages 212–218, Copyright © 2009 Spanish General Council of Optometry. Published by Elsevier España, S.L. All rights reserved. published with permission from Elsevier España, S.L.).

The variations in the PC in changing light conditions can dramatically affect the centration during ablation (Figures 3, 4, 5). The PC shifts in different light conditions relative to CSCLR. Erdem et al. [33] evaluated the location and shift of the PC relative to the coaxially sighted corneal reflex on horizontal and vertical planes under natural and pharmacologically dilated conditions in 94 (64 myopic and 30 hyperopic) eyes of 47 patients. The mean distance between the PC and the coaxially sighted corneal reflex was greater in hyperopes than in myopes (P < 0.05), but no significant difference was observed in PC shifts between myopes and hyperopes under all three conditions (P > 0.05). They concluded that the PC is located temporally and shifts in every direction, primarily infero-temporally, relative to the coaxially sighted corneal reflex with natural and pharmacologic dilation.

Since the PC is a non-stable target, a morphological reference is more advisable in refractive surgery. de Ortueta et al. [34] proposed the use of the corneal vertex (CV) measured by a videokeratoscope as a morphological reference to center corneal refractive procedures.

de Ortueta and Schreyger [35] evaluated a method for centering the ablation in standard hyperopic LASIK using an excimer laser with a video-based eye tracker system. They shifted the ablation centration from the PC to the vertex normal of the cornea using pupillary offset measured with the Keratron Scout videokeratoscope. They analyzed outcomes of 52 consecutive hyperopic eyes treated with the ESIRIS excimer laser, 3 months postoperatively and found that a refractive outcome of <0.50 D of spherical equivalent was achieved in 94% (49/52) of eyes with no eye losing more than one line of best spectacle-corrected visual acuity (BSCVA).

#### Hybrid centration approaches

Schruender et al. [36] presented a method to measure the three-dimensional shape of the cornea and to use the data for registration purposes in order to optimize ablation pattern alignment during corneal laser surgery. They measured the three dimensional shape of the cornea with a modified fringe projection technique using UV laser pulses. They used the peripheral elevation data (which is not affected during the laser treatment) for registration.

Arba-Mosquera et al. [37] described a method for centering ablation profiles considering PC and CV information simultaneously. They developed novel ablation profiles to cover the pupil aperture while respecting the CV as the optical axis of the ablation [asymmetric offset (AO)]. Their idea was to combine higher order aberrations (HOAs) referred to the PC (LOS) with manifest refraction values referred to the CV (visual axis). The ablation volume of AO profiles lies between the ablation volumes of no offset and symmetric offset ablation profiles. When combined with HOAs, AO ablation profiles affect specific HOA terms. Asymmetric offset spherical components affect HOA coma components, and AO astigmatic components affect HOA trefoil components. Further clinical studies are needed to support their theoretical results. This method should specially benefit non-coaxial eyes with large angle kappa (or alpha and lambda). Due to the smaller angle kappa associated with myopes compared to hyperopes, centration issues are less apparent. However, the angle kappa in myopes can be sufficiently large to show differences in results.

A summary of the findings regarding the various centration metrics is presented in Table 2.

**Table 2:** **Centration parameters of the human eye reported by various research groups**

**Parameters**	**Pande et.al. [** 3 **]**	**Erdem et al. [** 26 **]**			**Chan CC et al. [** 28]			**Yang Y et al. [** 54 **]**
**PC from VA (mm)**	0.75 (max) 0.34 ± 0.20 temp	-			-			-
**CR from VA (mm)**	0.62 (max)0.021 ± 0.16 nas	-			-			-
**GCC from VA (mm)**	1.06 (max)0.55 ± 0.22 temp	-			-			-
**PC to CR (mm)**	-	Pho0.336 ± 0.181 (temp)	Mes:0.345 ± 0.195(temp)	Dil:0.339 ± 0.170 (temp)	Pre-op:0.34 ± 0.24 (nas)	1 D post-op: 0.10 (temp)	3 M post-op: 0.07 (temp)	
**Mean magnitude of PC shift (mm)**	-	Mes to Pho: 0.084 ± 0.069	Pho to Dil: 0.149 ± 0.080	Mes to Dil: 0.102 ± 0.104	-			Mes to Pho: 0.133

Dil: pharmacologically dilated conditions; Mes: mesopic; Pho: photopic; PC: entrance pupil center; GCC: geometrical corneal center; CR: coaxially sighted corneal reflex; VA: corneal intercept of visual axis; temp: temporal; nas: nasal; D: day; M: month.

### Comparative studies between different centration methods

A summary of the comparative studies between different centration methods is presented in Table 3.

**Table 3:** **A summary of the comparative studies between different centration methods**

**Study**	**Laser platform**	**Sub-group**	**n**	**Condition**	**Follow up**	**Pre-op MRSE (D)**	**Post-op MRSE (D)**	**Res. Ref. (D)**	**BCVA**	**Saf index**	**Eff index**
**Corneal light reflex**											
**Okamoto et al. [** 38 **]**	NIDEK CXIII	CSCLR	268	Myo	1 M	−4.88 ± 1.55	0.17 ± 0.39		-	1.18	1.047
		LOS	288	Myo	1 M	−5.05 ± 1.63	0.19 ± 0.48		-	1.138	0.997
**Wu et al. [** 39 **]**	AOV	CLR	60	-	-	-	-	90% within ±0.5 (Astig)	No significant difference (p > 0.05)	-	-
		PC	59	-	-	-	-	72.8% within ±0.50.5 (Astig)		-	-
**Okamoto et al. [** 40 **]**	Nidek	CSCLR	317	Myo	3 M	-	+0.123 ± 0.378	-	-	Significantly higher for CSCLR (P < 0.05)	
		LOS	269	Myo	3 M	-	+0.187 ± 0.480	-	-		
**Visual Axis**											
**Kermani et al. [** 42 **]**	Nidek	LOS	181	Hyp	3 M	+2.46 ± 1.32	+0.19 ± 0.57	64% within ±0.5	92% within 1 line	-	-
		Visual Axis	64	Hyp	3 M	+2.57 ± 1.26	+0.29 ± 0.70	81% within ±0.5	91% within 1 line	-	-
**Normal corneal vertex**											
**Arbelaez et al. [** 4 **]**	SCHWIND	CV	24	Myo Astig	6 M	-	-	-	38% improved	-	-
		PC	29	Myo Astig	6 M	-	-	-	24% improved	-	-

CSCLR: coaxially sighted corneal light reflex; CLR: corneal light reflex; LOS: line of sight; CV: corneal vertex; PC: pupil center; n: number of eyes; Myo: myopia; Hyp: hyperopia; Astig: astigmatism; MRSE: manifest refraction spherical equivalent; BCVA: best corrected visual acuity; Res. Ref.: residual refraction; saf Index: safety index; eff Index: efficacy index.

#### Favoring corneal light reflex

Okamoto et al. [38] compared refractive outcomes of myopic LASIK with centration on the CSCLR to centration on the center of the pupil (LOS). For the CSCLR group, the laser ablation was delivered 80% closer to the visual axis. In decimal notation, the safety index (mean postoperative BSCVA/mean preoperative BSCVA) and efficacy index (mean postoperative UCVA/mean preoperative BSCVA) were statistically significantly higher in the CSCLR group compared to the LOS group (P < 0.05). This trend was accentuated in a subgroup analysis of patients with >0.25 mm difference between the CSCLR and LOS, favoring the CSCLR group. A statistically significantly greater induction of higher order aberrations (P = 0.04) and coma (P < 0.01) was noted in the LOS group postoperatively. They concluded that myopic LASIK centered on the CSCLR was significantly safer and more effective than LASIK centered on the pupil (LOS), with significantly lower induction of coma and total higher order aberrations.

Wu et al. [39] evaluated the clinical efficacy of LASIK (using the AOV Excimer laser) with ablation centration on the corneal optical center (corneal light reflex) using standard sphero-cylindrical ablation model. Treatments were divided into 2 groups: the experimental group with ablation centered on the corneal optical center and the control group with ablation centered on the PC. The distance between ablation center and CV normal was measured to describe the matching of ablated tissue and virgin cornea. The mean value was 0.35 ± 0.15 mm in the experimental group versus 0.69 ± 0.23 mm in the controls, and the difference between the two groups was significant (P < 0.05). The increase of root mean square of HOAs was smaller in the experimental group (P < 0.01), as compared to the control group. They concluded that the corneal optical center is a superior ablation reference compared to PC.

Okamoto et al. [40] compared refractive outcomes, HOAs, and CS of myopic wavefront-guided aspheric LASIK centered on the CSCLR or on the LOS, using the optical path difference customized aspheric treatment (OPDCAT) algorithm and the Navex excimer laser platform (both Nidek Co., Ltd.). Data at 3 months were compared based on the distance (P-distance) between the CSCLR and the LOS. Each group (CSCLR and LOS) was divided in three subgroups: high-distance subgroup (P-distance greater than 0.25 mm), intermediate-distance subgroup (P-distance greater than 0.15 mm and less than 0.25 mm) and low distance subgroup (P-distance less than 0.15 mm). The HOAs (P < 0.001) and coma (P = 0.001) were significantly higher in the LOS group. The LOS group had a significantly greater change in CS (P = 0.026). The centration on the CSCLR resulted in better safety, effectiveness, and CS than LOS centration.

#### Favoring light of sight

Bueeler et al. [41] determined the shifts of the main corneal reference points in relation to the chosen centration axis for the treatment. They performed computer simulations on several variations of the Gullstrand-Emsley schematic eye modified by an off-axis fovea. The postoperative LOS was found to depend least on the choice of the preoperative centration axis for both myopic and hyperopic treatments. It undergoes a maximum movement of 0.04 mm when centering a +5.0 D correction on the preoperative LOS, whereas the corneal reflex, which is used for centering most topography systems, can move by more than 0.1 mm. They concluded that centration of the correction on the preoperative LOS enabled good comparability between preoperative and postoperative measurements that use the LOS as a reference axis. Yet, centration of the treatment on the preoperative LOS does not ensure comparability between preoperative and postoperative measurements that use the corneal reflex as a reference axis like most corneal topography systems.

#### Favoring visual axis

Kermani et al. [42] reported refractive outcomes of hyperopic LASIK with automated centration on the visual axis compared with centration on the LOS. The NIDEK Advanced Vision Excimer Laser platform (NAVEX) was used to treat eyes with centration on the LOS (LOS group) and the visual axis (visual axis group). The coordinates of the visual axis were digitally transferred to the excimer laser system based on the positional relationship between the LOS and the CSCLR. Their initial experience with hyperopic LASIK centered on the visual axis indicated safe and predictable outcomes.

#### Favoring normal corneal vertex centration

Arbelaez et al. [4] compared the clinical outcomes of “aberration-free™” ablation profiles based on the normal CV and the PC in relation to LASIK using the SCHWIND platform. “Aberration-free™” aspheric ablation treatments were performed in all cases. Two myopic astigmatism groups were included: CV centered using the offset between PC and normal CV and PC centered using the PC. Induced ocular coma was on average 0.17 μm in the CV group and 0.26 μm in the PC group (comparison CV/PC, P = 0.01, favoring CV). Induced ocular spherical aberration was on average +0.01 μm in the CV group and +0.07 μm in the PC group (comparison CV/PC, P = 0.05, favoring CV). Change in asphericity was on average +0.56 in the CV group and +0.76 in the PC group (comparison CV/PC, P = 0.05, favoring CV). They concluded that in myopic eyes with moderate to large pupillary offset, CV-centered treatments performed better in terms of induced ocular aberrations and asphericity, but both centrations were identical in photopic VA.

### Discussion

The techniques of refractive surgery are evolving with the ongoing research. Studies [43,44] on subjects with normal vision have revealed that high VA is not related to perfect optics or any particular HOA. The parabolic approximation of the Munnerlyn algorithm has been studied in relation to an increase in corneal asphericity [45]. The ablation profiles have been optimized to compensate for the loss of ablation efficiency at non-normal incidence [46-49] along with the customization in optimum Zernike terms for minimum tissue ablation and time [50-53]. Thermal controls ensure the minimization of thermal load on the cornea to protect from tissue denaturation [54-57]. Active eye tracking during the refractive procedure and transformation algorithms aid the transformation of Zernike eye aberration coefficients for scaling, rotation and translation in the pupil [58-60]. An eye tracker makes the laser beam follow the eye movements and helps avoid severe decentration, however, studies show that an active eye-tracking system alone cannot ensure good centration [61]. Patient cooperation and fixation are important. Changes in the location of the PC with changes in the dilation of the pupil are typically slight, but can be significant in a few subjects, especially in pharmacologically dilated pupils. Yang et al. [62] found that the PC shifted consistently temporally as the pupil dilated. The total motion was relatively small, with a mean distance of 0.133 mm motion between the mesopic and photopic conditions, with the pupil diameter changing from 6.3 to 4.1 mm. Netto et al. [63] revealed an inverse correlation between the pupil size and age, but there was no relationship with gender or level of refraction. Guirao et al. [64] studied the effect on image quality expected when an ideal correcting method translates or rotates with respect to the pupil. They computed the residual aberrations that appear as a result of translation or rotation of an otherwise ideal correction. Based on their obtained analytical expressions, they provided practical rules to implement a selective correction depending on the amount of decentration. They suggest that typical decentrations only slightly reduce the optical benefits expected from an ideal correcting method. Benito et al. [65] found that after hyperopic LASIK, because of induction of negative spherical aberration and change in coma, disruption of the compensation mechanism leads to a larger increase of ocular aberrations. Comastri et al. [66] gave selection rules for the direct and inverse coefficients’ transformation and analyzed the missing modes associated with certain displacement directions. Taking these rules into account, they presented a graphical method to qualitatively identify the elements of the transformation matrix and their characteristic dependence on pupil parameters. The lateral alignment accuracy needed in wavefront-guided refractive surgery to improve the ocular optics to a desired level in normally aberrated eyes has been quantified. Bueeler et al. [67] found that to achieve the diffraction limit in 95% of the normal eyes with a 7.0 mm pupil, a lateral alignment accuracy of 0.07 mm or better was required. An accuracy of 0.2 mm was sufficient to reach the same goal with a 3.0 mm pupil.

Another interesting aspect of ocular aberrations was explored by Tran et al. [68]. They measured and compared the changes in objective wavefront aberration and subjective manifest refraction after LASIK flap creation with a mechanical microkeratome and a femtosecond laser. Their results led to a conclusion that the creation of the LASIK flap alone can modify the eye’s optical characteristics in low-order aberrations and HOAs. A significant increase in HOAs was seen in the microkeratome group, but not in the femtosecond laser group. This may have significant clinical implications in wavefront-guided LASIK treatments, which are based on measurements (corneal, ocular or based on ray tracing) made before flap creation. In another study [69], better astigmatic outcomes with the IntraLase laser were observed compared to microkeratome assisted refractive surgery.

Cyclotorsion in the seated and the supine patient has been measured in many studies. Statistical significance of cyclotorsion on the visual outcomes after refractive surgery has been argued in the past [70]. The rotational movement of the eye can influence any centration reference to a certain degree. Furthermore, the relationship between the vertex and pupil centration can also vary during rotation. This can affect the ablations designed by converting the axis centration reference in comparison to the reference followed in the diagnostic devices.

Fang et al. [23] studied the influence of treatment decentration and especially that of the transition zone (TZ) on induced wavefront aberrations. They found that the TZ played a significant role in the influence of decentration on the induced aberrations (mainly coma and spherical aberrations) in refractive surgery.

Artal et al. [71] found that in most young eyes, the amount of aberrations for the isolated cornea is larger than for the complete eye, indicating that the internal ocular optics (mainly the crystalline lens) play a significant role in compensating for the corneal aberrations thereby producing an improved retinal image. This compensation is larger in the less optically centered eyes that mostly correspond to hyperopic eyes, suggesting a type of mechanism in the eye’s design that is the most likely responsible for this compensation. They found that the distribution of aberrations between the cornea and lens appears to allow the optical properties of the eye to be relatively insensitive to variations arising from eye growth or exact centration and alignment of the eye’s optics relative to the fovea. These results may indicate the presence of an auto-compensation mechanism that renders the eye’s optics robust despite large variations in ocular shape and geometry. Similar findings have been reported by other authors [72-75]. Juan et al. [76] found horizontal coma compensation to be significantly larger for hyperopic eyes where angle kappa also tended to be larger. They proposed a simple analytical model of the relationship between the corneal coma compensation effect with the field angle and corneal and crystalline shape factors. They showed that the eye behaves as an aplanatic optical system, an optimized design solution rendering stable retinal image quality for different ocular geometries. In general, the angle alpha, kappa and lambda tend to be higher with increasing hyperopia. Therefore, finding the offset and the differences between the different optical neural axes is rather easy for high hyperopes, moderately easy for low hyperopes, moderately difficult for low myopes, yet very difficult for high myopes. For the minority of high myopic cases presenting with a large offset, consideration of the offset while centering the ablation can strongly influence the success and failure of the treatment.

The difference between the entrance and actual pupil size implies that any corneal irregularity or scarring overlaying the entrance pupil will cause irregular refraction and glare. For a glare-free vision, the OZ of the cornea must then be larger than the entrance pupil. In conventional LASIK treatment using the Alcon LADARVision 4000 platform, a larger surgical OZ diameter was found to significantly decrease HOAs after LASIK [77].

Arba-Mosquera et al. [78] analyzed the theoretical impact of decentered ablations in inducing coma. They found theoretically, that “aberration-free™” profiles should be centered referred to corneal apex, whereas customized treatments should be centered according to the diagnosis reference (since the aberrations maps are described for a reference system in the entrance PC). Ideally, customized, wavefront guided treatments should be measured with respect to the CSCLR and subsequent ablations centered on the CSCLR. They further stated that main HOA effects (coma and spherical aberration) result from the edge effects, strong local curvature change from OZ to TZ, and from TZ to non-treated cornea. Hence, it is necessary to emphasize the use of large OZs (covering scotopic pupil size), and smooth TZs.

In a study by Applegate et al. [79], two key principles emerged. First, the aberrometer’s measurement axis must coincide with the eye’s LOS. Second, the videokeratographer’s measurement axis (the vertex normal) must be parallel with the eye’s LOS. When these principles are satisfied, the eye will be in the same state of angular rotation and direct comparison of measurements is justified, provided any translation of the pupil from the vertex normal is taken into account. The error incurred by ignoring pupil displacement in videokeratography varies between eyes and depends on the type of aberration and amount of displacement, with the largest residual correction root-mean-square wavefront error being 1.26 μm over a 6.0 mm pupil, which markedly decreases retinal image quality. In another study, the videokeratography procedure has been tested to permit estimation of the corneal wave aberration from videokeratoscopic data with an accuracy of 0.05-0.2 μm for a pupil 4–6 mm in diameter [80].

Recently, Arba Mosquera and Verma [81] proposed a simple and inexpensive numerical (nonwavefront-guided) algorithm to recenter the OZ and to correct the refractive error with minimal tissue removal. Based on the reconstruction of ablation achieved in the first surgical procedure, they calculated a target ablation (by manipulating the achieved OZ) with adequate centration and an OZ sufficient enough to envelope the achieved ablation. The net ablation map for the retreatment procedure is calculated from the achieved and target ablations and is suitable to expand, recenter, and modulate the lower-order refractive components in a retreatment procedure. The results of their simulations suggest minimal tissue removal with OZ centration and expansion. Enlarging the OZ implied correcting spherical aberrations, whereas inducing centration implied correcting coma. Guirao et al. [82] presented a method for optimizing the correction of the eye’s higher-order aberrations in the presence of decentrations. They derived analytical expressions to estimate the fraction of every aberration term that should be corrected for a given amount of decentration and found that partial correction is more robust compared to complete correction.

With a myriad of clinical studies on refractive and ocular surgery based on different centration techniques presented here, a confusion and difference of opinion is bound to arise over choosing a favorable method. Similarly, the optical aberrations of the eye could be calculated and measured with different referencing, but a standard is imperative to be consistent and have a common language within the community. An optical society association (OSA) taskforce formed at the 1999 topical meeting on vision science and its applications [83] decided upon the standards for reporting the optical aberrations of eyes. The committee recommended that the ophthalmic community use the LOS as the reference axis for the purposes of calculating and measuring the optical aberrations of the eye (second by subcommittee of OSA [84]). The rationale was that the LOS in the normal eye is the path of the chief ray from the fixation point to the retinal fovea. Therefore, aberrations measured with respect to this axis will have the PC as the origin of a Cartesian reference frame. Since the exit pupil is not readily accessible in the living eye whereas the entrance pupil is, the committee recommended that calculations for specifying the optical aberrations of the eye be referenced to the plane of the entrance pupil. The committee also recommended that the instruments be designed to measure the optical properties of the eye and its aberrations be aligned co-axially with the eye’s LOS. If another reference axis is chosen for diagnosis, it must be converted to the standard reference axis using conversion formulas. However, such conversions should be avoided since they involve measurement and/or estimation errors for the two reference axes (the alignment error of the measurement and the error in estimating the new reference axis).

## Conclusion

Defining the optimum center for laser ablation is difficult with many available approaches, each of them claiming to provide good results. The problem comes from the fact that the real cornea is not a rotationally symmetrical volume, and the human eye is an asymmetrical optical system [85]. Usually, ablations are designed with three different centration references that can be detected easily and measured with currently available technologies (pupil centration/LOS and CSCLR).

PC may be the most extensively used centration method for several reasons. First, the pupil boundaries are the standard references observed by the eye-tracking devices. Moreover, the entrance pupil can be well represented by a circular or oval aperture, similar to the most common ablation areas. Centering on the pupil offers the opportunity to minimize the OZ size (and hence ablation depth and volume). However, OZ should be the same size or slightly larger as the functional entrance pupil for the requirements of the patient to avoid post-operative quality of vision symptoms such as glare, haloes, and starbursts [86,87]. Further HOAs arise from edge effects, i.e. strong local curvature changes from the OZ to the TZ, and from the TZ to the untreated cornea. For a patient who fixates properly, the PC defines the LOS (which is the reference axis recommended by the OSA for representing the wavefront aberrations). But the PC is not necessarily the reference for which the patient is actually driving the visual axis during manifest refraction. More importantly, the PC is unstable and changes with the pupil size. Therefore, a more morphological reference is advisable and in this case, the CSCLR.

If the human optical system were truly coaxial, CV (defined as the point of maximum elevation) would represent the corneal intercept of the optical axis. Ray tracing indicates that the optical axis is the ideal centering reference. Despite the fact that the human optical system is not truly coaxial, the cornea is the main refractive surface. Thus, CV represents a stable preferable morphologic reference. CV can be determined from the CSCLR (1st Purkinje image) and is used widely in refractive surgery. Small aperture intracorneal inlays have also shown better outcomes when centered referencing the CSCLR [88]. Tabernero and Artal [89] calculated the monochromatic and polychromatic Strehl ratios as a function of the pinhole position in 16 personalized eye models using actual data. They found that in eyes with little astigmatism and aberrations, the optimum centration of the small aperture were near the corneal reflex position. In their opinion, some small residual myopia and correction of corneal astigmatism might be required to improve optical outcomes with the inlay. The optimum centration depends on the type of corneal inlay. For an artificial pupil inlay, centration reference to the smallest possible pupil (i.e. strong lights on, but natural pupil) should be preferred while for refractive inlays, CSCLR should be preferred to avoid coma and trefoil.

The CSCLR can be considered as non-constant, but is dependent on the direction of gaze of the eye with respect to the light source. Furthermore, for a higher angle kappa, the corneal reflex can result in perceived coma induction as HOAs are measured with respect to the PC with aberrometers. Therefore, ablations centered using the pupillary offset, have the distance between the PC and the normal CV advocated. It must be noticed that on the less prevalent oblate corneas, the point of maximum curvature (corneal apex) might be off-center and not well represented by the CV. In those cases, PC is probably more stable. Both PC (LOS referenced) and CV (CSCLR referenced) centered ablations have presented clinical success, however the popular evidence favors CSCLR. The use of pupillary offset and asymmetric offset for centration reference, is gaining popularity in recent times. In theory, even under the consideration of the SC-effect and wide-field vision (as opposed to on axis foveal vision), an ideal OZ covering the widest entrance pupil is imperative to avoid glare and has shown to result in improved clinical outcomes [90]; this may be as important as the centration reference. The reduction in potential optical side effects of axis misalignment with a wider total treatment zone is at the cost of increased tissue consumption, however, low and moderate corrections usually present with enough tissue to remain within safety limits. Therefore, typical total treatment zones today range between 6.5 mm and 9.0 mm. Safety margins are necessary, but clinical practice encounters feasibility of high-end precision versus relevance of potential visual symptoms.

## Abbreviations

LOS: Line of sight
CV: Corneal vertex
TCA: Transverse chromatic aberration
VA: Visual acuity
PC: Pupil center
OPD: Optical path difference
CS: Contrast sensitivity
OZ: Optical zone
CSCLR: Coaxially sighted corneal light reflex
LASIK: laser in situ keratomileusis
BCVA: Best corrected visual acuity
BSCCS: Best spectacle-corrected contrast sensitivity
BSCVA: Best spectacle-corrected visual acuity
AO: Asymmetric offset
HOA: Higher order aberrations
OPDCAT: Optical path difference customized aspheric treatment
TZ: Transition zone
